# Evaluation of Self‐Mediated Alternatives for Risk Testing Education and Return of Results ‐ eSMARTER: A randomized trial from the Alzheimer’s Prevention Initiative program

**DOI:** 10.1002/alz70859_099986

**Published:** 2025-12-25

**Authors:** Carolyn Langlois, Angela R. Bradbury, Elisabeth Wood, Kristin Harkins, Claire M Erickson, Hayley Salata, Trisha L Walsh, Emily A. Largent, Brian L Egleston, Eric M. Reiman, Joshua D Grill, J. Scott Roberts, Nicholas J. Ashton, Jason Karlawish, Jessica B. Langbaum

**Affiliations:** ^1^ Banner Alzheimer's Institute, Phoenix, AZ USA; ^2^ University of Pennsylvania, Philadelphia, PA USA; ^3^ University of Pennsylvania Perelman School of Medicine, Philadelphia, PA USA; ^4^ Fox Chase Cancer Center, Philadelphia, PA USA; ^5^ Institute for Memory Impairments and Neurological Disorders, University of California, Irvine, Irvine, CA USA; ^6^ University of Michigan School of Public Health, Ann Arbor, MI USA; ^7^ Banner Sun Health Research Institute, Sun City, AZ USA; ^8^ Banner Alzheimer's Institute and University of Arizona, Phoenix, AZ USA

## Abstract

**Background:**

Availability of amyloid modifying therapies dramatically increases the need to disclose Alzheimer’s disease (AD) related genetic and/or biomarker test results to inform clinical decision making. An existing shortage of genetic counselors and dementia specialists, coupled with the 21st Century Cures Act requirement to immediately return most medical test results, makes timely development of scalable methods to responsibly communicate test results essential.

**Method:**

The Evaluation of Self‐Mediated Alternatives for Risk Testing Education and Return of Results (eSMARTER) is a decentralized, randomized, non‐inferiority trial evaluating if disclosure of APOE genotype by participant‐directed scalable digital platforms is non‐inferior to telehealth disclosure with a healthcare provider. Participants also have the option to learn plasma pTau‐217 test results. eSMARTER is enrolling approximately 600 adults aged 60‐80 who have undergone prior APOE testing but report not knowing their APOE results. Participants are randomized to receive results either with via telehealth with a healthcare provider or independently through a digital intervention. Participants in the digital arm can select a web‐based intervention or a chatbot. Participants complete online assessments evaluating cognitive, affective and behavioral patient reported outcomes prior to and at various timepoints after disclosure. Blood samples for pTau‐217 testing and repository storage are collected using remote mobile phlebotomy services. Strategically timed newsletters support participant retention and engagement. Additionally, eSMARTER participants are invited to the ancillary eDROP‐AD study to assess feasibility and acceptance of self‐sampling at‐home using finger prick blood collection with the Capitainer^®^SEP10 card.

**Result:**

Enrollment in eSMARTER began in October 2024 and is expected to close in February 2025. Recruitment, screening, and enrollment data will be presented along with participant demographics and key health‐related characteristics. Unique insights and lessons learned from launching a national, decentralized, randomized trial will also be shared.

**Conclusion:**

The eSMARTER study addresses the need for practical and scalable methods to responsibly communicate AD genetic and/or biomarker results. The eSMARTER digital platforms will be made available to support healthcare providers in returning AD genetic and/or biomarker results to individuals in clinical and research settings. All data and samples collected will be made available for public sharing.

